# The Surgical Club of South West England

**Published:** 1983-10

**Authors:** 


### The Surgical Club of South West England

Abstracts of Papers given at the Spring Meeting 29–30 April 1983 at Yeovil

### UPPER GI CANCER

R. J. Clarke, Yeovil

Carcinoma of the oesophagus and stomach are known to carry a poor prognosis. Surgical treatment, particularly of the oesophagus is difficult and carries a high mortality. Since it is difficult to separate gastric from oesophageal carcinoma, all the gastric and oesophageal carcinomas referred to one surgeon over a 10 year period are reviewed.

One hundred and thirty six patients have been included, 72 purely gastric carcinomas and 64 with carcinoma either originating in or involving the oesophagus.

In only 32 of the 72 gastric carcinomas was gastrectomy possible. Of these 32 patients 2 (6%) died postoperatively and only 4 patients survived more than 5 years. Of 64 carcinomas involving the oesophagus (23 squamous and 41 adenocarcinoma) oesophagogastrectomy was possible in only 43; 9 (21%) of these 43 patients died postoperatively and only 2 patients survived more than 5 years; 2 (4.3%) of these patients leaked from the oesophageal anastomosis. No patient undergoing palliative oesophageal intubation survived 1 year.

The results were assessed according to both stage of progression and grade of tumour. Only those tumours which were well differentiated and of limited spread had any reasonable hope of cure, although many patients had worthwhile palliation by resection.

The place of adjuvant therapy and earlier diagnosis are discussed to see if the depressing prognosis can be improved.

### THE SAFETY OF IMMEDIATE RESECTION FOR OBSTRUCTING CARCINOMA OF THE DISTAL COLORECTUM

D. R. Mackrell and N. J. Barwell, Truro

The traditional management of obstructing tumours of the left colon or rectum has been emergency transverse colostomy followed by delayed resection and colostomy closure. Methods to reduce the cumulative morbidity and mortality of the 3-stage approach include immediate resection with a protective stoma and 1-stage excision of the tumour and dilated proximal colon, with ileo sigmoid or ileorectal anastomosis. To determine the relative safety of these options, 56 patients (mean age 70.3 yr) admitted to this hospital between 1977–82 with obstructing carcinomas distal to the mid-transverse colon were reviewed. Of 52 patients with resectable cancers, 17 had initial colostomy alone (10 came to later resection), 18 had subtotal colectomy with anastomosis and 17 had less extensive primary resections, 4 with unprotected anastomoses. The choice of operation was mainly dictated by consultant preference. Patients receiving any type of immediate resection had a lower overall mortality (8.6%) than those with initial colostomy alone (35.3% : $P = 0.025$). Subtotal colectomy carried a mortality of 11%. For survivors of the staged procedure the length of hospital stay (mean 38.5 days) was much greater than for subtotal colectomy (22.4 : $P = 0.005$). In treating obstructing carcinoma of the distal colorectum, primary resection is at least as safe as colostomy alone. Subtotal colectomy nearly halves the duration of in-patient care without increased risk.

### TEACHING AIDS IN THE OUTPATIENT DEPARTMENT

D. A. Griffiths, Yeovil

The early years of postgraduate training for the acute clinical specialities takes place in the larger teaching centres where the trainee is expected to teach as well as learn. Most career clinicians work in non-teaching establishments where the systematic training of the medical specialities is overshadowed by the demands of the ward, outpatients and operating theatres. Medical education continues in these establishments and the classes include patients, nurses, ancillary staff, general practitioners and junior doctors. This paper deals with various methods used to educate this wider audience in the clinical environment of the district hospital. The aids range from written instructions, printed diagrams, paper models, audio-tapes to disposable plastic anatomical models. The whole body can be covered with these aids and they serve as a semi-permanent reminder of diagnosis and therapy.

In conclusion, inexpensive disposable aids can be developed from many materials to teach patients about their condition. The physician with an interest in medical education will inevitably reap the benefit by having better informed patients and staff.

### EXTERNAL FIXATION FOR MAJOR LIMB INJURY

John Tricker, Yeovil

Major limb injury with neurovascular complications and loss of soft tissue is now a common orthopaedic problem. Bone stability can be achieved by using external fixators which allow access to the soft tissue for later repair. The first external fixator for clinical use was designed by Dr. Alvin Lambot in Paris in 1902. This device held the fracture by introducing skeletal pins into the bone on either side of the fracture and connecting these pins by an external solid rod. All the external devices used today have evolved from this apparatus.

A short history of the development and design of these external fixators was given and a résumé of the common types of fixator available today with their individual advantages and disadvantages was discussed.

### MODERN AVIATION MEDICINE

B. Pingree Surg. Cdr. R.N. Air Stn. Yeovilton

The role of the Royal Naval Air Station at Yeovilton was outlined and operational medical and physiological hazards defined. Attention was drawn to the importance of the advent of the pressure cabin in aircraft. This development overcame such traditional problems of aviators as hypoxia, decompression sickness and cold. Methods of escape from stricken aircraft were reviewed and the incidence of vertebral crush fracture from ejection seats was shown to be of the order of 50%. This figure had not improved despite the more recent introduction of rocket assisted ejection seats. Developments in cockpit instrumentation were discussed particularly the 'head up' and more recent 'head down' displays which present information on aircraft configuration and systems. The applicability of this technology to the achievement of increased realism in flight simulators was noted. Of more direct clinical interest, a trend towards reduced exclusivity on medical grounds was observed, with conditions such as treated hypertension and past pneumothorax being compatible with the holding of a commercial pilot's licence under defined circumstances. Continuing developments to improve aircrew helmets, clothing and equipment assemblies were anticipated. Finally some preliminary medical lessons and aviation experiences from the recent Falklands Campaign were presented. Organisation and casualty management were found to be effective and appropriate to the resources available to the Task Force.

### PELVIC RADIOTHERAPY, REVIEW OF CURRENT LITERATURE

M. K. Teoh, Yeovil

Chronic complications may appear from as early as two months or as late as five years. Work by De Cosse in 1969 and others since then have identified several factors which are important in the development of post-radiation complications. These are, dosage, technique, previous pelvic surgery, arteriosclerosis, hypertensions and diabetes.

The underlying pathology is ischaemia and the characteristic histological features include increased submucosal fibrosis, arteriosclerosis, lymphatic ectasia and endarteritis obliterans.

Most series report an incidence of around 10% following pelvic irradiation. The incidence of large and small bowel complications are roughly equal and 70% had more than one complication, the most common being strictures. Generally proctocolitis, rectal ulcers and steatorrhoea tend to occur later than perforation, fistulae and strictures. There is a high mortality rate of around 35% from these complications.

The management of these bowel complications can be difficult. In small bowel damage primary resection should be extensive but even so the anastomotic leakage rate is 36% in one large series. Bypass procedures carry a lower leakage and mortality rate.

A defunctioning colostomy is usually adequate in large bowel problems. A permanent colostomy may be the best way to treat rectovaginal fistulae.

The management of ureteric obstruction caused by post-radiation periureteral fibrosis is controversial. Ureteral dilatation and ureteroneocystostomy both give disappointing results. Ileal substitution and ileal conduits when utilising only the proximal ureter achieve far better results. Transverse colon conduit and cutaneous ureterostomy have been tried successfully but numbers studied were small.

### EXPERIENCE WITH CELESTIN TUBE INTUBATION AT YEOVIL DISTRICT HOSPITAL

**Arun Nigam, Yeovil**

A study of 31 patients was made who had undergone Celestin tube intubation at Yeovil Hospital from 1976 to 83. Seventy-four percent of patients had carcinoma of the oesophagus and 26% had benign strictures associated with peptic oesophagitis. Out of the malignant lesions 70% were in males and 30% were in females. The majority of patients were in the age group between 60 and 80 years. The complications associated with the Celestin tube intubation and the quality of life was judged from the hospital records and direct interviews.

The most frequent complication was bronchopneumonia (48%). This was seen both in the early stages and as a late complication. Wound infection was seen in 19% of cases. During the similar period 149 oesophageal dilatations were carried out for benign strictures. There were three perforations giving an incidence of 2%. Two were treated with celestin tube intubation while one succumbed.

Displacement of tubes was seen in four cases (13%). Three were proximal displacements requiring endoscopic replacement in two cases. In one case the tube displaced distally, 17 months after intubation for a benign stricture and had migrated to the sigmoid colon where it perforated. There were 16 separate perforations in the small bowel. The patient survived surgery and is alive and well. The incidence of blocked tubes was also 13%. Three patients required repeat oesophagoscopy to unblock them and two had replacement tubes. In the third case it unblocked itself by using 1 : 20 hydrogen peroxide. Haematemesis was seen in four patients while one developed empyema.

Over half the patients had died within 3 months of intubation. The longest survival was in those with benign strictures. The quality of life as judged by improvement in swallowing, gain in weight and frequency of blockage of tubes was found to be good to fair in most patients. The symptom of dysphagia was much improved.

### 'SOLDIERS ON EVEREST'

**P. J. Horniblow, Yeovil**

This was an account of the successful joint British–Nepalese Army Expedition to Everest in the pre-monsoon period of 1976. Dr. Horniblow conducted his audience from Khatmandu to Namche Bazaar, showing photographs of the beautiful terrain through which expeditions have passed since John Hunt's successful party showed the way in 1953. He drew attention to the valuable acclimatisation afforded by the 180 mile walk-in and the role played by both Sherpas and Sherpanis as cheerful and willing porters. Once base camp was established at 18,000 feet at the foot of the Ice Fall from the Western Cwm, the build up of the high camps proceeded smoothly, despite the tragic death of Captain Terry Thompson, R.M., who was killed in a crevasse. The lecturer showed the unusually treacherous condition of the Ice Fall in 1976, due to the marked absence of snow the preceding winter, exposing the ice to the direct rays of the sun. He apologised for the crude surgical skill he displayed in treating the severely prolapsed piles of a Gurkha corporal at Camp 3, at 22,000 feet, but assured the audience that the patient was walking, albeit with difficulty, when he met him again in Khatmandu a month later. He also described the frost-bite incurred by the summit pair, Sergeant Lane and Corporal Stokes, which resulted in multiple amputations later that year, and recalled how his fellow doctor, Colonel Dick Hardy, R.A.M.C., had set an altitude record for the over-40's by reaching Camp 6, at over 26,000 feet.

### SURGEONS AND SURGERY IN VICTORIAN YEOVIL

**Revd J. R. Guy, Archivist, Yeovil District Hospital**

Yeovil District Hospital opened as a Dispensary in 1858. Its founders were three local surgeons, E. T. Warry, a pupil of Abernethy at Bart's; Russell Aldridge; and W. F. Bennet, who had served with Florence Nightingale at Scutari.

C. D. Steele, a Yeovil surgeon of the 1850s, had described the extraction of loose cartilages from the elbow joint under chloroform in 1854, and the use of gold-beater's skin in the wound closure. George Flower (1880) had operated in 1895 in a private house on a case of dislocation of the femur onto the pubes, fracture of the neck, and removal of the head of the bone. Edward Garland (surgeon YDH, 1864–93) described a case of dermoid ovarian tumour, escaping per rectum, and Ptolemy Colmer, his successor, the appearance of a pin in the appendix of a $7\frac{1}{2}$ year old child.

W. A. Hunt (surgeon-dentist Y.D.H., 1869–1904) was the first to use cocaine by hypodermic injection as a local anaesthetic in dental extraction in England. He was a pioneer of dental anaesthesia, who rose to be President of the B.D.A. His father, dental surgeon to the orginal dispensary, was a friend of James Robinson, the London dentist who first used ether in England as a general anaesthetic for tooth extraction two days before Liston's historic surgical procedure. Robinson communicated his experience to William Hunt senior in Yeovil, who was the first provincial dentist in the country to adopt the practice.

